# Predicting the WHO/ISUP Grade of Clear Cell Renal Cell Carcinoma Through CT-Based Tumoral and Peritumoral Radiomics

**DOI:** 10.3389/fonc.2022.831112

**Published:** 2022-02-14

**Authors:** Yanqing Ma, Zheng Guan, Hong Liang, Hanbo Cao

**Affiliations:** ^1^ Cancer Center, Department of Radiology, Zhejiang Provincial People’s Hospital, Affiliated People’s Hospital, Hangzhou Medical College, Hangzhou, China; ^2^ The Department of Radiology, Hangzhou Medical College, Hangzhou, China

**Keywords:** radiomics, clear cell renal cell carcinoma, computed tomography, peritumor, WHO/ISUP grade

## Abstract

**Objectives:**

This study aims to establish predictive logistic models for the World Health Organization/International Society of Urological Pathology (WHO/ISUP) grades of clear cell renal cell carcinoma (ccRCC) based on tumoral and peritumoral radiomics.

**Methods:**

A cohort of 370 patients with pathologically confirmed ccRCCs were included in this retrospective study between January 2014 and December 2020 according to the WHO/ISUP grading system. The volume of interests of triphasic computed tomography images were depicted manually using the “itk-SNAP” software, and the radiomics features were calculated. The cohort was segmented into the training cohort and validation cohort with a random proportion of 7:3. After extraction of radiomics features by analysis of variance (ANOVA) or Mann-Whitney *U* test, correlation analysis, and the least absolute shrinkage and selection operator (LASSO) method, the logistic models of tumoral radiomics (LR-tumor) and peritumoral radiomics (LR-peritumor) were developed. The LR-peritumor was subdivided into LR-peritumor-2mm, LR-peritumor-5mm, and LR-peritumor-10mm, and the LR-peritumor-2mm was subdivided into LR-peritumor-kid and LR-peritumor-fat based on the neighboring tissues of ccRCCs. Finally, an integrative model of tumoral and peritumoral radiomics (LR-tumor/peritumor) was built. The value of areas under the receiver operator characteristics curve (AUCs) was calculated to assess the efficacy of the models.

**Results:**

There were 209 low-grade and 161 high-grade ccRCCs enrolled. The AUCs of LR-tumor in CT images of venous phase were 0.802 in the training cohort and 0.796 in the validation cohort. The AUCs were higher in the LR-peritumor-2mm than those in LR-peritumor-5mm and LR-peritumor-10mm (training cohort: 0.788 vs. 0.788 and 0.759; validation cohort: 0.787 vs. 0.785 and 0.758). Moreover, the AUCs of LR-peritumor-fat were higher compared with those of LR-peritumor-kid. The LR-tumor/peritumor displayed the highest AUCs of 0.812 in the training cohort and 0.804 in the validation cohort.

**Conclusions:**

The tumoral and peritumoral radiomics helped to predict the WHO/ISUP grades of ccRCCs. On the diagnostic performance of peritumoral radiomics, better results were seen for the LR-peritumor-2mm and LR-peritumor-fat.

## Introduction

Renal cell carcinoma (RCC) predominated in all renal malignancies, accounting for 90% of all cases (1). Clear cell renal cell carcinoma (ccRCC) is the most frequent subtype encountered in RCCs with 70%–80% incidence, causing a worse prognosis and significant mortality (2). According to the fourth edition of the World Health Organization/International Society of Urological Pathology (WHO/ISUP) grading system (3), the RCCs were classified into grades I to IV. The prognosis, therapy strategy, and routine surveillance relied on tumor pathological grades (4). Partial nephrectomy has been recommended as the preferred choice for the management of locoregional renal tumors (2). The visceral fat is a biologically active tissue that contributes to chronic inflammation and releases numerous adipokines which have been reported to strongly promote tumor initiation and progression (5). A meta-analysis of adherent perinephric fat concluded that it leads to an increased blood loss and a prolonged operating time (6). It has been reported that the radiomics model combining tumoral and peritumoral radiomics features was of most significance in distinguishing renal angiomyolipoma and ccRCC (7). Therefore, understanding its tumoral and peritumoral peculiarity such as perirenal fat thickness (8) has assumed great significance in clinical and pathological research, as well as in treatment decisions (2).

Radiomics is a computer-assisted approach that transforms conventional medical images into high-throughput quantitative digital features (9) and tries to explore the association between radiomics features with pathological heterogeneity (10) and clinical outcomes (11). To date, several studies have indicated that a radiological logistic model based on computed tomography (CT) radiomics features can predict the WHO/ISUP grades of ccRCCs (12) and the preoperative magnetic resonance imaging (MRI) radiomics signature can effectively predict the tumor size, stage, grade (13), and necrosis score of ccRCCs (14). In addition, CT-based peritumoral radiomics is also a promising way to predict its malignancy grades (15). However, far too little attention has been paid to the implication of radiomics analysis in perinephric fat and the role of integrative tumoral and peritumoral radiomics. To the best of our knowledge, this is the first study that focused on tumoral and peritumoral radiomics and subdivided the fat component from the peritumoral tissue to analysis.

In this study, we carefully subsected the peritumoral tissue into peritumoral kidney (peritumor-kid) and peritumoral fat (peritumor-fat) and detailed its radiomics manifestations of CT images to identify the change of tumoral and different peritumoral tissues. We then integrated the tumoral and peritumoral radiomics features to predict the WHO/ISUP grades of ccRCCs.

## Materials and Methods

This retrospective study was approved by the institutional ethics committee of our hospital (No. 2020QT181), and the consents of patients were waived.

### Patient Population

**Figure 1:**
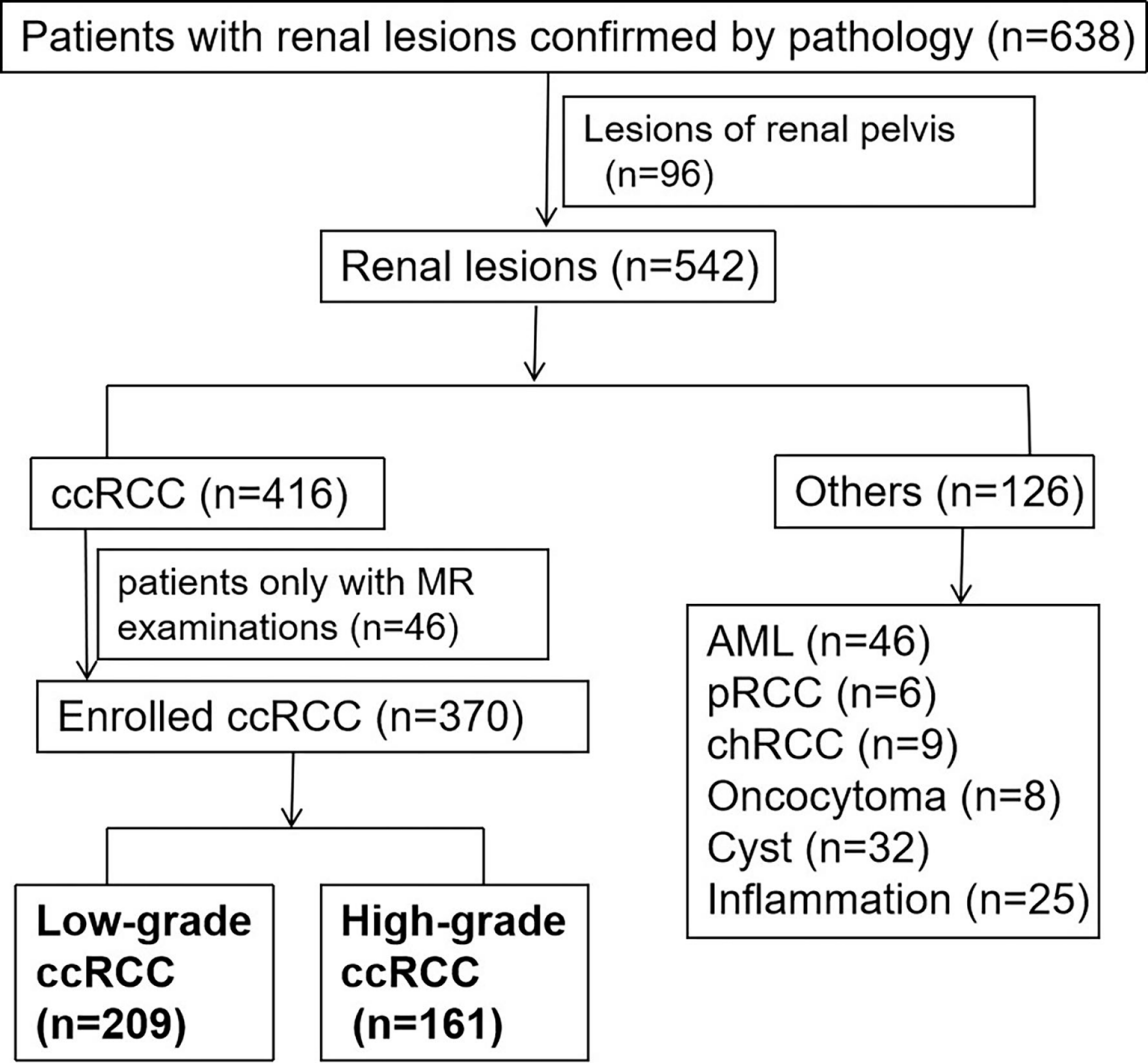
The flowchart of patient enrolment. Finally, there were 209 low-grade patients and 161 high-grade patients selected.

After searching the pathological database of our institution between January 2014 and December 2020, a total of 370 patients were eligible (**Figure 1**). The inclusion criteria were as follows: (a) confirmed by surgery and pathology to be ccRCC; (b) taken triphasic CT examinations including unenhanced phase, arterial phase, and venous phase; and (c) the CT examinations were proceeded within 2 weeks before surgeries. The exclusion criteria were as follows: (a) had more than one masses in the kidney; (b) the lesion is entirely located inside the kidney which was not adjacent to the peritumor-fat tissue; and (c) had received chemotherapy or radiotherapy before surgeries.

As the existing research recognized that a two-tiered grading into low grade and high grade for ccRCCs provided similar survival information compared with the WHO/ISUP grading system (16), therefore, in this study, we referred grades I and II ccRCCs as low-grade tumors and grades III and IV as high-grade ones. Finally, there were 209 ccRCC cases of low grade and 161 of high grade ones.

### CT Examination

Three hundred and seventy patients have taken CT examinations by a 64-slice (133 patients) or 128-slice (237 patients) spiral CT scanner (Siemens, Somaton Definition AS, Munich, Germany). Triphasic CT examination was performed, and the parameters were as follows: tube voltage 120 kVp, tube current 150–200 mA, collimation 64 * 0.625 mm, rotation time 0.75 s, slice thickness 5 mm, interval thickness 5 mm, and nonionic contrast media iomeperol at a rate of 3.0 ml/s with a dose of 1.3 ml/kg. A 100-Hu threshold of the abdominal aorta at the celiac artery was taken as the baseline, then the arterial phase and venous phase were examined 15 and 30 s after the unenhanced phase.

### Volume of Interests Segmentation

**Figure 2:**
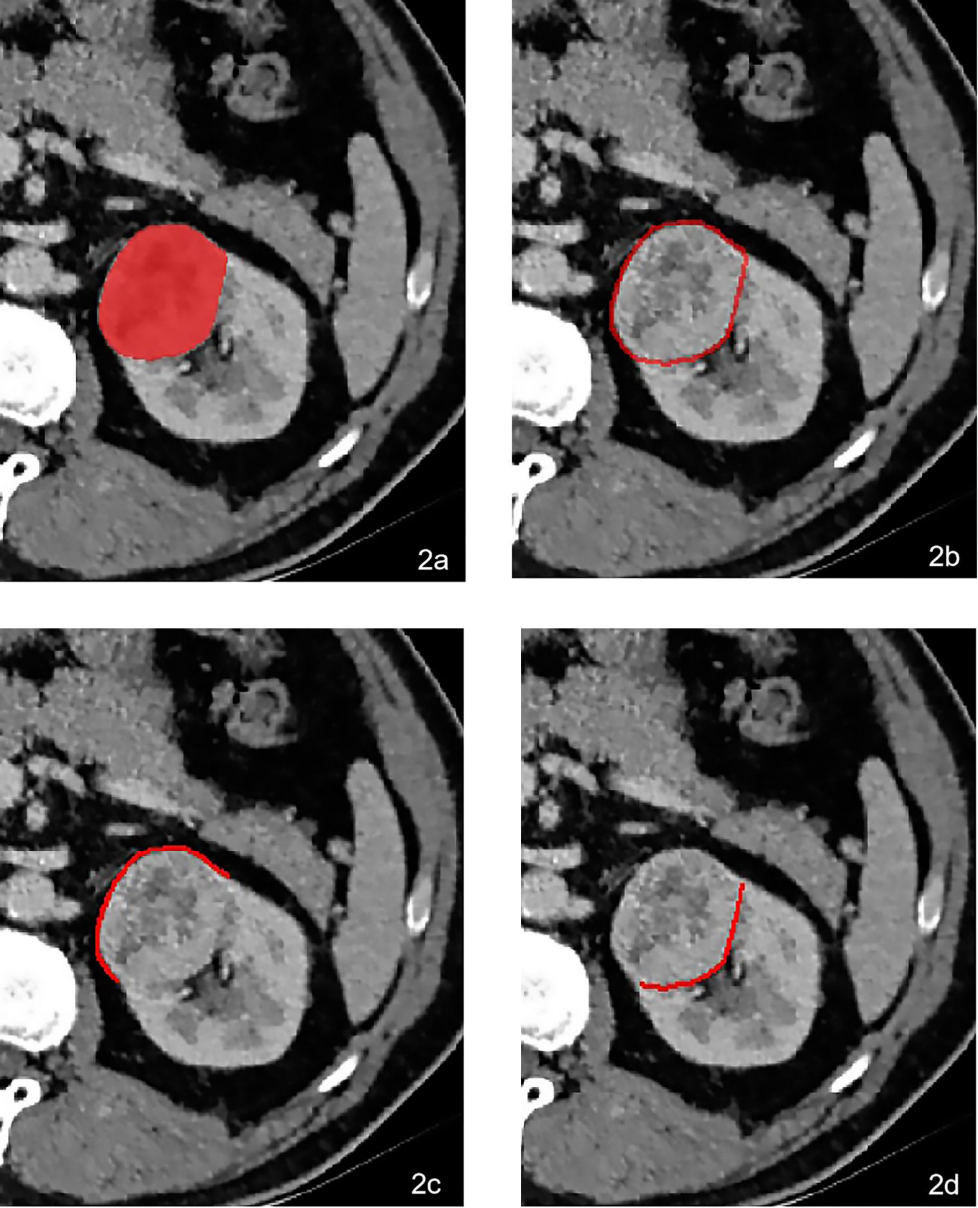
A 64-year-old male was pathologically confirmed to be high-grade ccRCC in the left kidney. The VOI was delineated manually in **(A)**. The peritumoral VOI was then received after expending 2 mm automatically in AK software **(B)**. The peritumoral VOI was subdivided into peritumor-fat **(C)** and peritumor-kid **(D)** according to the different neighboring tissues.

Firstly, the origin CT images were standardized automatically in “AK” software (Artificial Intelligence Kit, GE Healthcare, Chicago, IL, USA) after performing reconstruction of the voxel of X, Y, and Z into 1.0 mm, correcting the image gray lever into 1-32, and implementing Gaussian distribution. All the volume of interests (VOIs) of the tumors were then manually delineated in “itk-SNAP” software (https://www.itksnap.org/) by two radiologists with 7 and 10 years of experience (**Figure 2A**). Thirdly, the VOIs of the peritumor were achieved by the AK software after automatically expanding 2 mm (**Figure 2B**), 5 mm, and 10 mm outside the lesion contour, respectively. Lastly, the VOIs of peritumor-fat (**Figure 2C**) and peritumor-kid (**Figure 2D**) were divided manually based on the different interfaces neighboring the tumor.

### Radiomics Feature Extraction

#### The Radiomics Feature Calculation

After the segmentation of VOIs, the tumoral and peritumoral radiomics features were automatically calculated in AK software.

#### The Reproducibility of Radiomics Features

The radiomics features from two radiologists of unenhanced phase, arterial phase, and venous phase were compared using the intraclass correlation coefficient (ICC) method; the ICC greater than 0.75 was considered to be of good consensus between two observers.

#### The Data Balance

Due to the imbalance between two sample sets (209 low-grade ccRCCs and 161 high-grade ccRCCs), an approach of synthetic minority oversampling technique was performed to regulate the unbalanced ratio between two groups (17). The cohort was then divided into the training cohort with 146 low-grade and 113 high-grade ccRCCs and the validation cohort with 63 low-grade and 48 high-grade ccRCCs.

#### The Radiomics Feature Selection

ANOVA or Mann-Whitney *U* test, correlation analysis, and the LASSO method were used to extract the optimal radiomics features. A calibration method and the Homes-Lemeshow test were used to assess the goodness of fit of models. The specific process of radiomics analysis was elucidated in the **Supplementary Material**.

### Predictive Model Construction

The logistic model of tumoral radiomics features was developed (LR-tumor) after 100 bootstrapped replications to resample the data. The logistic models of peritumoral radiomics features including 2 mm (LR-peritumor-2mm), 5 mm (LR-peritumor-5mm), and 10 mm (LR-peritumor-10mm) peritumor models were constructed as well. Moreover, the analysis of peritumor was subdivided into peritumor-kid and peritumor-fat; the relevant logistic models of peritumor-kid (LR-peritumor-kid) and peritumor-fat (LR-peritumor-fat) radiomics were built. Finally, the logistic model combining tumoral and peritumoral radiomics features (LR-tumor/peritumor) was developed to predict the WHO/ISUP grade of ccRCCs. The Delong test was used to delineate the receiver operator characteristic curve (ROC). Also, the value of area under curve (AUC) with a 95% confidence interval (CI) was calculated to evaluate the efficacy of the corresponding model. The decision curve analysis (DCA) was used to quantify the net benefits at a range of threshold probabilities in the predictive model (18).

### Statistical Analysis

The R software (https://www.r-project.org/) was utilized to standardizing the origin images, balancing the sample sets, and selecting optimal radiomics features through Gaussian distribution, synthetic minority oversampling technique, ANOVA or Mann-Whitney *U* test, correlation analysis, LASSO method, DCA, and the Hosmer-Lemeshow test. The methods ICC and Delong test were carried out using the MedCalc software (https://www.medcalc.org/). In addition, the clinical demographic data were analyzed by means of independent *t*-test. A two-tailed *p*-value <0.05 was of statistical significance.

## Results

### The Analysis of LR-Tumor and LR-Peritumor

To the analysis of tumoral radiomic features, there were 9 optimal radiomics features left. The AUCs of LR-tumor were 0.802 (95% CI, 0.749–0.856) in the training cohort and 0.796 (95% CI, 0.711–0.881) in the validation cohort.

**Figure 3:**
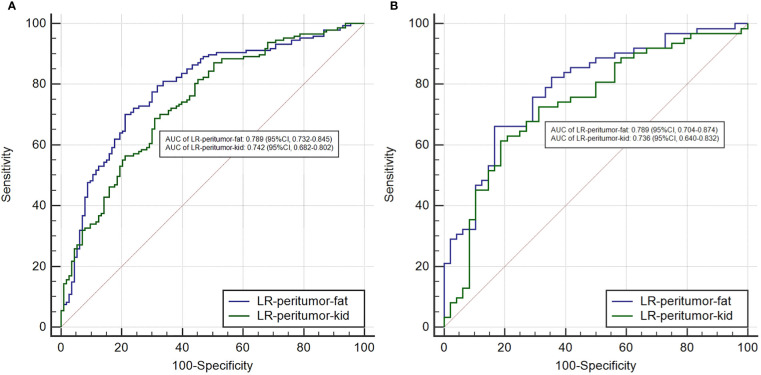
The comparison of AUCs between the LR-peritumor-fat and LR-peritumor-kid in the training cohort **(A)** and the validation cohort **(B)**.

To the analysis of peritumoral radiomics features, we compared the performance of different peritumoral contours of 2, 5, and 10 mm. Three peritumoral logistic models of LR-peritumor-2mm, LR-peritumor-5mm, and LR-peritumor-10mm were developed **Table 1**). The AUCs of LR-peritumor-2mm were relatively higher than those of LR-peritumor-5mm and LR-peritumor-10mm (training cohort: 0.788 vs. 0.788 and 0.759; validation cohort: 0.787 vs. 0.785 and 0.758). So we selected the peritumoral radiomics feature of 2 mm for further analysis. The peritumor based on the adjacent interface was subdivided into the peritumor-kid which was adjacent to the kidney and peritumor-fat which was adjacent to the perinephric fat. Subsequently, the LR-peritumor-kid and LR-peritumor-fat were constructed. The AUC of LR-peritumor-fat was 0.789 (95% CI, 0.732–0.845) in the training cohort and 0.789 (95% CI, 0.704–0.874) in the validation cohort, higher than those of the LR-peritumor-kid with 0.742 (95% CI, 0.682–0.802) in the training cohort (**Figure 3A**) and 0.736 (95% CI, 0.640–0.832) in the validation cohort (**Figure 3B**).

**Table 1:** The AUCs of tumoral and peritumoral radiomic models.

	Training cohort (95% CI)	*p*	Validation cohort (95% CI)	*p*
LR-tumor	0.802 (95% CI, 0.749–0.856)	<0.001	0.796 (95% CI, 0.711–0.881)	<0.001
LR-peritumor				
LR-peritumor-2mm	0.788 (95% CI, 0.733–0.843)	<0.001	0.787 (95% CI, 0.697–0.877)	<0.001
LR-peritumor-5mm	0.788 (95% CI, 0.732–0.843)	<0.001	0.785 (95% CI, 0.699–0.872)	<0.001
LR-peritumor-10mm	0.759 (95% CI, 0.700–0.817)	<0.001	0.758 (95% CI, 0.665–0.851)	<0.001
LR-peritumor-2mm				
LR-peritumor-kid	0.742 (95% CI, 0.682–0.802)	<0.001	0.736 (95% CI, 0.640–0.832)	<0.001
LR-peritumor-fat	0.789 (95% CI, 0.732–0.845)	<0.001	0.789 (95% CI, 0.704–0.874)	<0.001
LR-tumor/peritumor	0.812 (95% CI, 0.759–0.864)	<0.001	0.804 (95% CI, 0.720–0.888)	<0.001

The AUCs of different tumoral and peritumoral radiomics models. The AUC was calculated by Delong test, and the value of p < 0.05 indicated statistical significance.

### The Integrative Analysis of LR-Tumor/Peritumor

**Figure 4:**
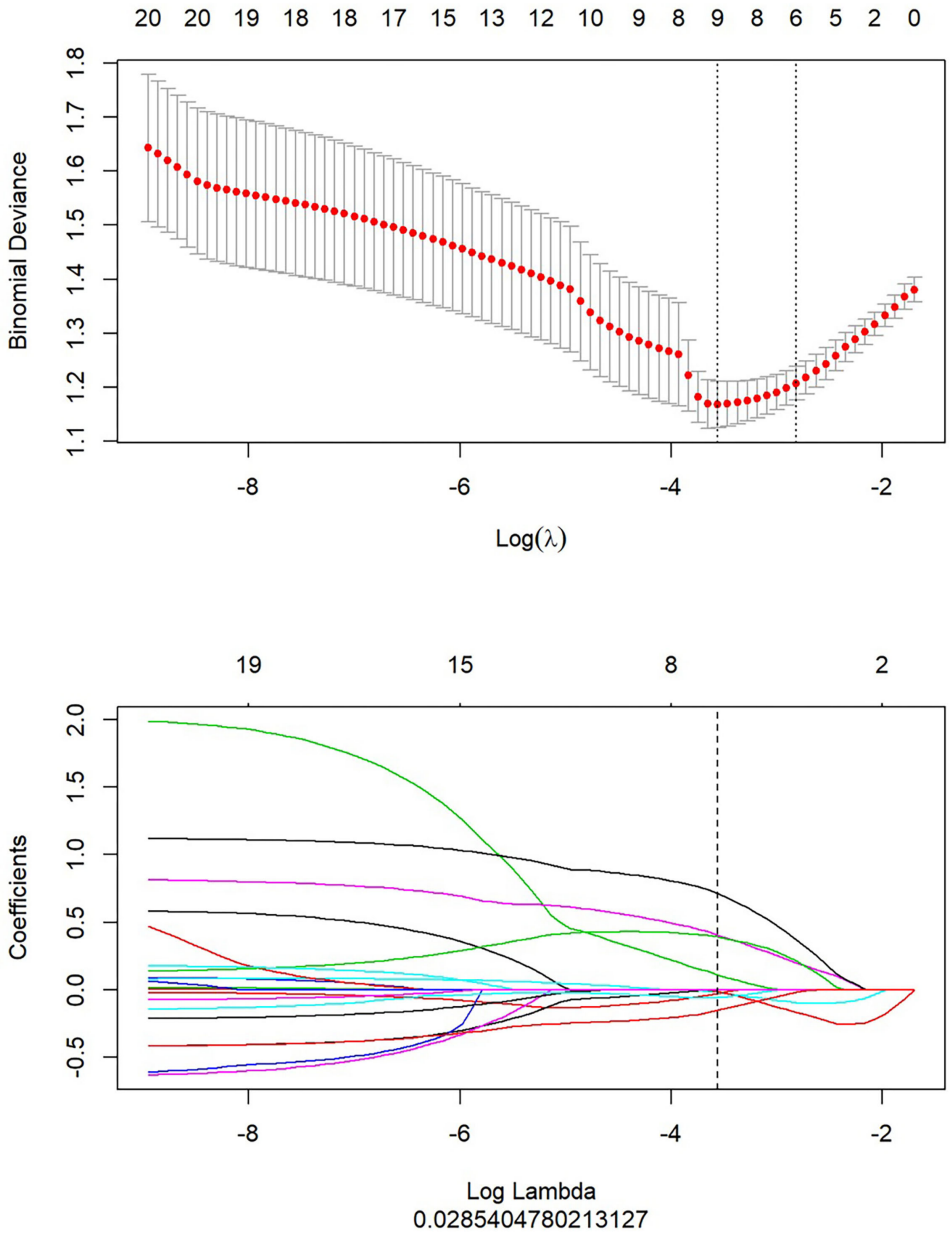
After extraction of radiomics features, there were 9 optimal radiomics features left to constitute the LR-tumor/peritumor.

**Figure 5:**
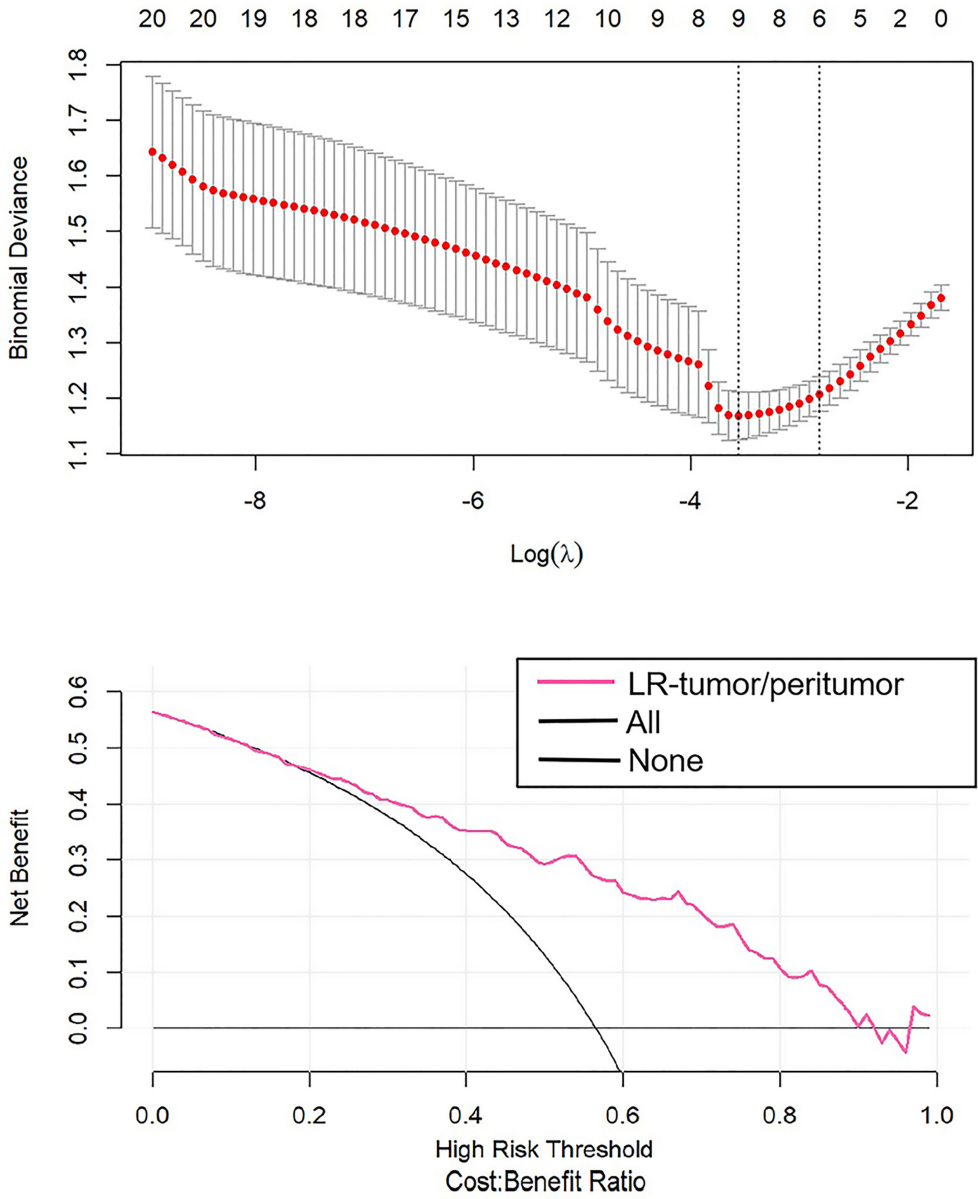
The DCA curve showed the different net benefits at a range of threshold probabilities in LR-tumor/peritumor.

**Figure 6:**
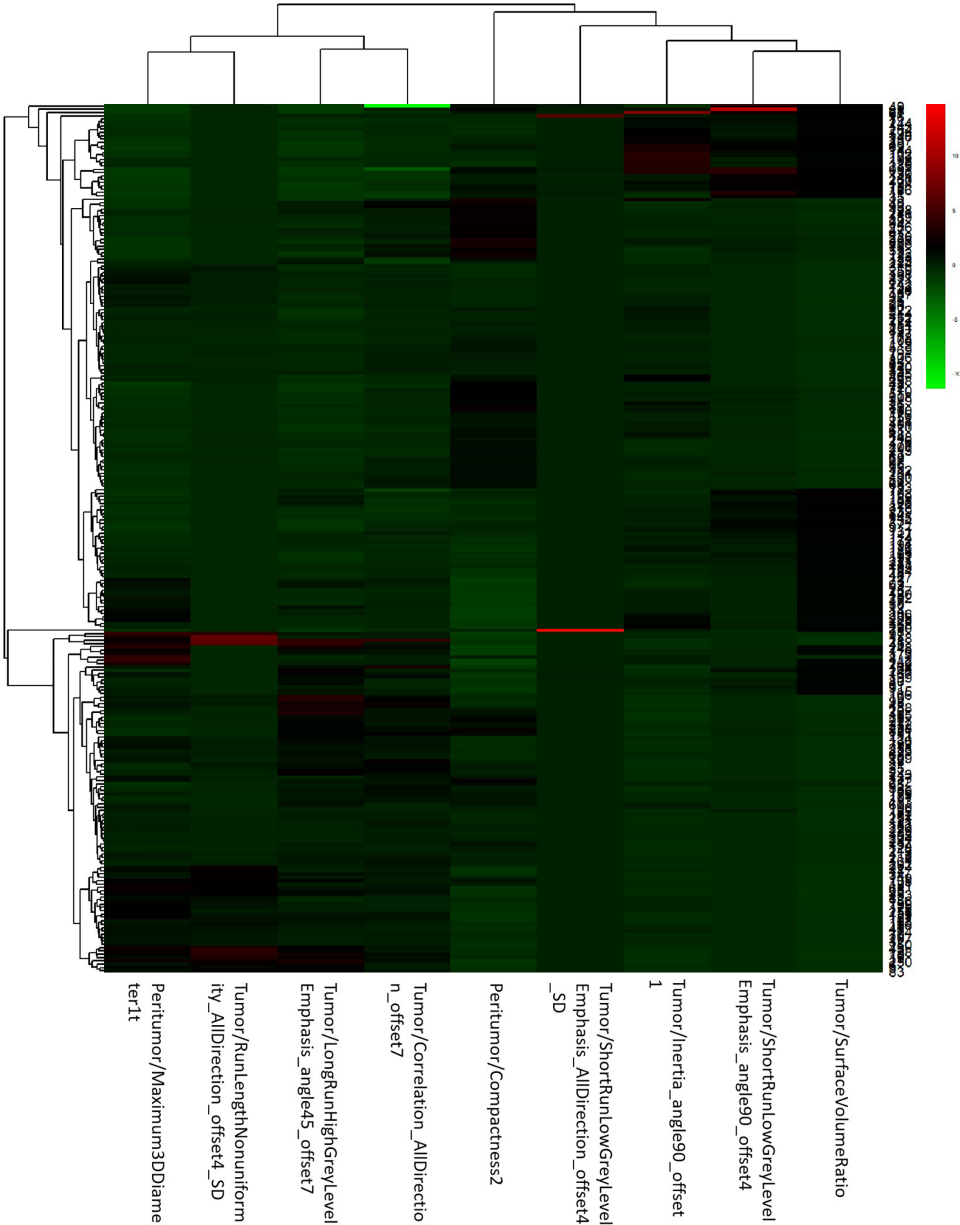
The heat map of LR-tumor/peritumor included 9 selected radiomics features.

After the study of simple tumoral and peritumoral radiomics, the integrative analysis collaborating these two factors was conducted. The AUC of LR-tumor/peritumor was 0.812 (95% CI, 0.759–0.864) in the training cohort and was 0.804 (95% CI, 0.720–0.888) in the validation cohort. There were 9 radiomics features extracted to form this logistic model after the least absolute shrinkage and selection operator method (**Figure 4**). The identification efficacy of LR-tumor/peritumor was better than that of the LR-tumor and LR-peritumor alone. The DCA curve illustrated in **Figure 5** showed the accuracy of this model. The heat map including 9 radiomics features is illustrated in **Figure 6**.

### Clinical Demographics

The baseline clinical demographics are listed in **Table 2**. There were 209 cases pathologically confirmed as low-grade ccRCCs with 57 females and 152 males; the mean age was 56.8 ± 12.6 years old. A total of 51 females and 110 males were incorporated in high-grade ccRCCs, and the mean age was 59.5 ± 12.6 years old. The variables of age (*p* = 0.038), location (*p* = 0.033), and long diameter (*p* = 0.000) had statistical difference. It seems that the high-grade ccRCCs were more likely to be located in the left kidney. The long diameter of high-grade ccRCCs was larger than its low-grade ones.

**Table 2:** General clinical characteristics.

	Low grade (*n* = 209)	High grade (*n* = 161)	*p*
Sex (female/male)	57 (27.3%)/152 (72.7%)	51 (31.7%)/110 (68.3%)	0.357
Age (mean ± SD)	56.8 ± 12.6	59.5 ± 12.6	0.038
Location (right/left)	113 (54.1%)/96 (45.9%)	69 (42.9%)/92 (57.1%)	0.033
Long diameter (mm)	33.7 ± 17.9	53.5 ± 23.1	0.000

The clinical variables of age, location, and long diameter had statistical difference. However, there was no statistical significance in terms of gender.

### Reproducibility of Triphasic CT Images

The ICC of triphasic CT radiomics features between two radiologists greater than 0.75 was of good agreement. After comparing radiomics features of ICCs greater than 0.75 in the unenhanced-phase, arterial-phase, and venous-phase CT images, 78.8% unenhanced-phase radiomics features matched, 86.6% arterial-phase radiomics features matched, and 89.1% venous-phase radiomics features matched. Therefore, the CT images of the venous phase were chosen and the mean values of radiomics features from two radiologists were calculated for the analysis.

## Discussion

In a previous study on the local advanced RCC, it has been demonstrated that its infiltrative radiological characteristics were significantly accompanied with larger tumor size and higher clinical T stage (19). Considering that radiological infiltrative characteristics correspond highly to pathological findings, we made attempts to explore the correlation between radiomics signature and pathological WHO/ISUP grades in ccRCCs. Our results also showed that high-grade ccRCCs appeared to have significantly longer diameter with a mean value of 53.5 ± 23.1 mm compared with low-grade ccRCCs with 33.7 ± 17.9 mm. Another significant difference was observed in the variables of age and location.

The literature on diffusion-weighted MRI of ccRCCs identified that perinephric fat invasion was an independent predictor of tumor pathological grades, demonstrating a likelihood of its higher aggressiveness (20). Thus, it is reasonable to add the debate on the performance of peritumoral radiomics. We inventively explored the peritumoral radiomics in three different ranges of 2, 5, and 10 mm. To the best of our knowledge, barely any study has speculated the peritumoral radiomics from different regions. The result suggested that LR-peritumor-2mm achieved a favorable prediction for the WHO/ISUP grades of ccRCCs, with a slightly higher AUCs compared with the LR-peritumor-5mm. The peritumor tissue of 2 mm was subdivided into peritumor-kid neighboring the normal kidney and peritumor-fat neighboring the adherent normal fat. The specific research of the peritumor radiomics revealed that the LR-peritumor-fat presented a better prediction of pathological grades than LR-peritumor-kid in ccRCCs (0.789 vs. 0.742 in the training cohort, 0.789 vs. 0.736 in the validation cohort). Our research demonstrated that peritumor-fat potentially impacted the WHO/ISUP grades of ccRCCs, which corroborated with other previous studies (21). As has been noted, adherent perinephric fat characterized by inflammatory adipose tissue influenced the outcomes of surgeries (22). It may be due to the malignancy nature of ccRCCs that causes chronic and systemic inflammation in the adjacent adipose tissue.

High-grade ccRCCs have a poorer prognosis compared with low-grade ccRCCs in terms of biologic behavior and prognostic factors which are related to pathological grades (23). Precisely predicting the pathological grades of ccRCCs through a noninvasive method of radiomics based on medical images is of great significance (24), allowing not only the assessment and characterization of ccRCCs but also the identification of patients with poorer prognosis who may benefit from early surveillance (25). We identified that the radiomics analysis of tumor and peritumor may aid in the preoperative prediction of the WHO/ISUP grades of ccRCCs with AUCs of 0.802 and 0.788 in the training cohort and 0.796 and 0.787 in the validation cohort. The integrative logistic model of LR-tumor/peritumor showed a preferred prediction in ccRCCs with AUCs of 0.812 and 0.804 in the training and validation cohorts. Our results were in line with the previous results that radiomics features are closely associated with biologic behaviors.

The study remains an area of significant limitations. First, since this is a single-center retrospective study, the generalization of these results requires independent validations. In addition, we simply subdivided the peritumor interface by 2 mm to analyze the radiomics of peritumor-kid and peritumor-fat. Thus, how the radiomics of different peritumor-kid and peritumor-fat varies is worthy of further study. Furthermore, we only assessed CT manifestations as it was the easiest-to-implement way to renal masses. In future research, a novel framework combined CT and MRI images should be extended to improve predictive efficiency. Thus, despite a large number of studies, radiomics still remains in a proof-of-principle concept.

Overall, we first report the evidence that peritumoral radiomics including peritumor-kid and peritumor-fat radiomics together with tumoral radiomics were significant predictive factors for the WHO/ISUP grades in ccRCCs. This readily available and noninvasive radiomics approach may help in the pathological grading of ccRCCs before surgeries.

## Data Availability Statement

The raw data supporting the conclusions of this article will be made available by the authors, without undue reservation.

## Ethics Statement

The studies involving human participants were reviewed and approved by the ethics committee of Zhejiang Provincial People’s Hospital. Written informed consent for participation was not required for this study in accordance with the national legislation and the institutional requirements.

## Author Contributions

YM: conceptualization, methodology, writing—original draft, writing—review and editing, and supervision. ZG: software, formal analysis, and data curation. HL: validation and resources. HC: resources and visualization. All authors listed have made a substantial, direct, and intellectual contribution to the work and approved it for publication.

## Funding

The research was funded by the fund of Medical and Health Research Project of Health Commission of Zhejiang Province (No. 2022492695).

## Conflict of Interest

The authors declare that the research was conducted in the absence of any commercial or financial relationships that could be construed as a potential conflict of interest.

## Publisher’s Note

All claims expressed in this article are solely those of the authors and do not necessarily represent those of their affiliated organizations, or those of the publisher, the editors and the reviewers. Any product that may be evaluated in this article, or claim that may be made by its manufacturer, is not guaranteed or endorsed by the publisher.
