# Supplementary material for: Predicting the WHO/ISUP Grade of Clear Cell Renal Cell Carcinoma Through CT-Based Tumoral and Peritumoral Radiomics
Source: Front Oncol. 2022 Feb 14;12:831112. doi: 10.3389/fonc.2022.831112 (PMC8884273; doi:10.3389/fonc.2022.831112)
Supplement: Supplementary file 1 [file DataSheet_1.doc]

Radiomics features includes histogram parameters, texture parameters, Gray level co-occurrence matrice (GLCM) parameters, gray level run-length matrice (GLRLM) parameters, and gray level Size Zone Matrice (GLZSM) parameters.

1. **Histogram parameters:** It concerns with properties of individual pixels. They describe the distribution of voxel intensities within the CT image through commonly used and basic metrics. It covers the followed parameters: Energy, Entropy, MaxIntensity, MinIntensity, Mean Value, Mean absolute deviation, MedianIntensity, Range ,Root mean square (RMS), Standard deviation (stdDeviation), Uniformity, Variance, Volume Count, Voxel Value Sum, Relative Deviation, Frequency Size, Quantiles, Percentiles, Skewness, and Kurtosis.
2. **Texture parameters:** It is one of the important characteristics used in identifying objects or regions of interest in an image, texture represents the appearance of the surface and how its elements are distributed. It is considered an important concept in machine vision, in a sense it assists in predicting the feeling of the surface (e.g. smoothness, coarseness …etc.) from image. It covers the followed parameters: Energy, Entropy, Correlation, Inertia, Cluster Shade, Cluster Prominence.
3. **Form Factor parameters:** It includes descriptors of the three-dimensional size and shape of the tumor region. It covered the followed parameters: Sphericity, Surface area, Compactness 1, Compactness 2, Inertia, Cluster Shade, Cluster Prominence.
4. **GLCM parameters:** It represents the joint probability of certain sets of pixels having certain gray level values. It calculates how many times a pixel with gray level i occurs jointly with another pixel having a gray value j. By varying the displacement vector d between each pair of pixels. It covers the followed parameters: Energy of GLCM, Entropy of GLCM, Inertia of GLCM, Correlation, Inverse Difference Moment, Haralick features. And the haralick features includes Haralick Correlation, Angular Second Moment, Contrast, Haralick Entropy, Hara Variance, sum Average, sum Variance, sum Entropy, difference Variance, difference Entropy, inverse Difference Moment.
5. **GLRLM parameters:** It is defined as the numbers of runs with pixels of gray level i and run length j for a given direction θ. RLMs is generated for each sample image segment having directions (0°, 45°, 90°&135°). It covers the followed parameters: Short/Long Run Emphasis, Gray Level Non-uniformity, Run Length Non-uniformity, Low/High Gray Level Run Emphasis, Short Run Low/High Gray Level Emphasis, Long Run Low/High Gray Level Emphasis.
6. **GLZSM parameters:** It is the starting point of Thibault matrices. For a texture image f with N gray levels, it is denoted GSf (s, g) and provides a statistical representation by the estimation of a bi-variate conditional probability density function of the image distribution values. It covers the followed parameters: Small/Large Zone Emphasis, Gray-level Non-uniformity, Zone-Size Non-uniformity, Zone Percentage, Low/High Gray-Level Zone Emphasis, Small Zone Low/High Gray-Level Emphasis, Large Zone Low/High Gray-Level Emphasis, Gray-Level Variance, Zone-Size Variance.

The method of radiomics signature comprises the following steps: Image standardization, VOI segmentation, intraclass agreement (ICC) analysis, and feature selection. The process of feature selection included feature proprecessing, analysis of variance (ANOVA)/Mann-Whitney U test (MW) dimensionality reduction, the correlation test, and the least absolute shrinkage and selection operator (LASSO). All of the above steps were carried out by R statistical software and the radiomics features were calculated by AK software.

1. **Image standardization**

We resampled all the images into a 1.0mm*1.0mm*1.0mm voxel size at X/Y/Z-spacing. Then denoising by Gaussian and normalizing image grey level to a scale from 1 to 32 were automatically proceed in software of AK (GE Healthcare).

1. **VOI segmentation**

3D volume of interest (VOI) were delineated by two radiologists with 7-10 years of CT diagnosis experience independently, in ITK-SNAP software. The volume of interests (VOIs) of the tumor were delineated by two radiologists with 7 and 10 years of experience, manually. And the VOIs of the peritumor were achieved by ”AK” software after automatically expanding 2mm, 5mm, and 10mm outside the lesion contour, respectively. Finally, the radiomics features were calculated on AK software, automatically.

1. **The intraclass agreement (ICC) analysis**

The intra-observer agreements of feature extraction were evaluated by the intraclass correlation coefficient (ICC). The ROI segmentation was performed by two experienced radiologists independently. Intra-observer ICC was computed by comparing extractions of reader A (with 7 years’ experience on abdominal MRI) and reader B (with 10 years’ experience on abdominal MRI). When the ICC was greater than 0.75, it was considered as good agreement and favorable extraction reproducibility. And the mean value of radiomics features from two radiologists were calculated as robust features for further analysis.

1. **Feature selection**
2. **Feature proprecessing**

We replaced the abnormal values by mean to standardize the data. Then, Partitioning the training set and testing set with proportion of 7:3. Thirdly, we used z-score normalization to make the image intensities have the properties of a standard normal distribution with$\mu\text{=}1$and$\sigma\text{=}0$, where$\mu$was the mean value of the images, and$\sigma$was the standard deviation. The normalized values (also called z scores) of the image intensities (*x*) were calculated as follows:

$$z=\frac{x-\mu}{\sigma}$$

1. **analysis of variance (ANOVA) and Mann-Whitney U test (MW)**

Firstly, after the normality test, the features without normal distribution were screened out. The the normal distributed features were dealed with variance homogeneity test, followed by independent t-test, and the features with greater contribution were selected. Secondly, all the features were re-analyzed by MW, the statistical significant features were selected. Finally, the features selceted by ANOVA and MW were merged.

1. **Correlation test**

The correlation test was calculated to reduce data redundancy. The software calculated the paired correlation between each two of the features. If the Spearman correlation coefficient was greater than 0.9, which showed that the two features were highly correlated, one of them was removed. All of the above steps were carried out by R statistical software version 4.0.1

1. **The least absolute shrinkage and selection operator (LASSO) algorithm**

LASSO is a powerful algorithm for regression analysis with high dimensional predictors. In our study, the LASSO algorithm was combined with the logistic regression model for model development. We used the LASSO logistic regression model to select the most important predictive features and construct a radiomics signature in the training set. This algorithm minimizes a log partial likelihood subject to the sum of the absolute values of the parameters bounded by a constant:


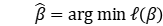
 , subject to
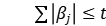


Where
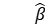
 is the obtained parameters,
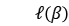
 is the log partial likelihood of the logistic regression model, and t >0 is a constant.

The LASSO algorithm shrinks some coefficients and reduces others to exactly 0 via the absolute constraint. Thus, LASSO is an outstanding method for feature selection by retaining the good features of both subset selection and ridge regression. In this study, LASSO selected 2 nonzero coefficients
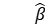
, and a formula was generated using a linear combination of selected features that were weighted by their respective LASSO coefficients. The “glmnet” package in R statistical software was used for LASSO logistic regression model analysis.

**The equation of LR-tumor**

Radscore= -0.857*SurfaceArea+0.117*ShortRunLowGreyLevelEmphasis_angle90_offset4-0.07*ShortRunLowGreyLevelEmphasis_AllDirection_offset4_SD+0.329*Inertia_angle90_offset1+0.03*SurfaceVolumeRatio-0.106*LongRunHighGreyLevelEmphasis_AllDirection_offset7_SD+0.206*HighGreyLevelRunEmphasis_AllDirection_offset7_SD+0.123*ShortRunEmphasis_angle0_offset4+0.257*LowIntensityLargeAreaEmphasis-0.029*LowIntensitySmallAreaEmphasis + 0.3

**The equation of LR-peritumor-2mm**

Radscore= 0.119*Maximum3DDiameter-0.002*Inertia_AllDirection_offset4_SD+0.106*RunLengthNonuniformity_AllDirection_offset1_SD+0.324*SurfaceArea+0.048*ClusterProminence_AllDirection_offset1_SD+0.024*HighIntensityLargeAreaEmphasis-0.315*Compactness2-0.247*LowGreyLevelRunEmphasis_AllDirection_offset4+0.127*ClusterShade_angle45_offset4-0.29

**The equation of LR-tumor/peritumor**

Radscore= -0.03*tumor/ShortRunLowGreyLevelEmphasis_AllDirection_offset4_SD+0.111*tumor/ShortRunLowGreyLevelEmphasis_angle90_offset4+0.406*tumor/Inertia_angle90_offset1+0.39*tumor/SurfaceVolumeRatio-0.02*tumor/LongRunHighGreyLevelEmphasis_angle45_offset7-0.014*peritumor/Maximum3DDiameter-0.058*tumor/RunLengthNonuniformity_AllDirection_offset4_SD+0.714*peritumor/Compactness2-0.152*tumor/Correlation_AllDirection_offset7 + 0.357
